# Supplementary material for: Emergence of Third-Generation Cephalosporin-Resistant Morganella morganii in a Captive Breeding Dolphin in South Korea
Source: Animals (Basel). 2020 Nov 6;10(11):2052. doi: 10.3390/ani10112052 (PMC7694518; doi:10.3390/ani10112052)
Supplement: Supplementary file 1 [file animals-10-02052-s001.zip › Supplements (Mm_animals)/Table S1. Mm(Fv).docx]

**Table S1**. The five prophage regions detected in the genome of *Morganella morganii* strain KC-Tt-01. The regions were determined by PHASTER (<http://phaster.ca/>) and are shown with their lengths, genomic locations, directions, and completeness.

| **Region (Length, G+C %)** | **Completeness^†^** | **Most common phage / BLAST Hit** | **E-Value** |
| --- | --- | --- | --- |
| **Region 1 (34.5kb, 50.15%)** | **Intact** | **PHAGE_Salmon_SEN5_NC_028701** |  |
| 1892..1919 |  | attL | 0 |
| complement(2055..3032) |  | PHAGE_Salmon_SEN5_NC_028701: integrase; PP_00004; phage(gi966201588) | 2.04E-143 |
| 3522..3794 |  | PHAGE_Salmon_SEN5_NC_028701: regulatory protein Cox; PP_00005; phage(gi966201593) | 1.93E-22 |
| 3876..4019 |  | hypothetical; PP_00006 | 0 |
| 4032..4427 |  | PHAGE_Salmon_Fels_2_NC_010463: hypothetical protein; PP_00007; phage(gi169936058) | 1.98E-06 |
| 4494..4715 |  | hypothetical; PP_00008 | 0 |
| 4708..4929 |  | PHAGE_Mannhe_phiMHaA1_NC_008201: DksA-like zinc finger domain containing protein; PP_00009; phage(gi109289947) | 1.73E-18 |
| 5197..5886 |  | PHAGE_Salmon_SEN5_NC_028701: methyl-directed repair DNA adenine methylase; PP_00010; phage(gi966201549) | 1.53E-48 |
| 5886..6209 |  | hypothetical; PP_00011 | 0 |
| 6206..8512 |  | PHAGE_Yersin_L_413C_NC_004745: gpA; PP_00012; phage(gi30065742) | 0 |
| 8646..9536 |  | hypothetical; PP_00013 | 0 |
| 9544..10470 |  | PHAGE_Citrob_vB_CfrM_CfP1_NC_031057: endoribonuclease; PP_00014; phage(gi100126) | 2.07E-131 |
| complement(10486..10650) |  | hypothetical; PP_00015 | 0 |
| complement(10875..11909) |  | PHAGE_Salmon_SEN5_NC_028701: portal vertex protein; PP_00016; phage(gi966201557) | 3.76E-140 |
| complement(11912..13630) |  | PHAGE_Salmon_SEN5_NC_028701: terminase ATPase subunit; PP_00017; phage(gi966201558) | 0 |
| 13777..14622 |  | PHAGE_Salmon_SEN5_NC_028701: capsid scaffolding protein; PP_00018; phage(gi966201559) | 1.89E-31 |
| 14635..15690 |  | PHAGE_Salmon_SEN5_NC_028701: major capsid protein; PP_00019; phage(gi966201560) | 5.78E-127 |
| 15721..16533 |  | PHAGE_Salmon_SEN5_NC_028701: terminase endonuclease subunit; PP_00020; phage(gi966201561) | 4.04E-68 |
| 16629..17114 |  | PHAGE_Salmon_SEN5_NC_028701: head completion-stabilization protein; PP_00021; phage(gi966201562) | 3.12E-36 |
| 17114..17314 |  | PHAGE_Salmon_SEN5_NC_028701: tail component protein; PP_00022; phage(gi966201563) | 1.16E-21 |
| 17317..17577 |  | hypothetical; PP_00023 | 0 |
| 17577..18122 |  | PHAGE_Entero_mEp237_NC_019704: lysin; PP_00024; phage(gi435439323) | 1.50E-89 |
| 18115..18621 |  | PHAGE_Erwini_PEp14_NC_016767: Rz protein; PP_00025; phage(gi374531879) | 3.14E-23 |
| 18618..18773 |  | hypothetical; PP_00026 | 0 |
| 18773..19189 |  | PHAGE_Salmon_SEN5_NC_028701: tail protein; PP_00027; phage(gi966201567) | 2.93E-38 |
| 19199..19840 |  | PHAGE_Salmon_SEN5_NC_028701: tail completion protein; PP_00028; phage(gi966201568) | 4.33E-57 |
| 19837..20466 |  | PHAGE_Salmon_SEN1_NC_029003: baseplate assembly protein V; PP_00029; phage(gi971764924) | 1.21E-75 |
| 20463..20801 |  | PHAGE_Erwini_ENT90_NC_019932: baseplate assembly protein; PP_00030; phage(gi431810974) | 1.41E-35 |
| 20804..21712 |  | PHAGE_Erwini_ENT90_NC_019932: baseplate assembly protein; PP_00031; phage(gi431810943) | 3.03E-146 |
| 21705..22313 |  | PHAGE_Escher_pro483_NC_028943: tail fibers protein; PP_00032; phage(gi971758530) | 1.94E-89 |
| 22310..23611 |  | PHAGE_Salmon_SEN5_NC_028701: tail fiber protein; PP_00033; phage(gi966201574) | 1.27E-68 |
| 23608..23961 |  | PHAGE_Edward_MSW_3_NC_020082: putative tail assembly chaperone; PP_00034; phage(gi448261067) | 1.07E-24 |
| complement(23933..24562) |  | PHAGE_Entero_HK106_NC_019768: tail fiber assembly protein; PP_00035; phage(gi428783304) | 4.92E-35 |
| complement(24563..25333) |  | PHAGE_Entero_HK97_NC_002167: tail fiber; PP_00036; phage(gi9634179) | 1.34E-24 |
| 25323..25889 |  | PHAGE_Entero_Mu_NC_000929: Gin; PP_00037; phage(gi9633542) | 5.55E-89 |
| complement(25925..26365) |  | PHAGE_Entero_P88_NC_026014: tail protein; PP_00038; phage(gi744692744) | 2.95E-62 |
| complement(26374..29259) |  | PHAGE_Entero_P88_NC_026014: tail tape measure protein; PP_00039; phage(gi744692745) | 0 |
| complement(29408..29707) |  | PHAGE_Salmon_SEN5_NC_028701: tail protein; PP_00040; phage(gi966201581) | 5.29E-18 |
| complement(29769..30281) |  | PHAGE_Salmon_SEN5_NC_028701: major tail tube protein; PP_00041; phage(gi966201582) | 1.76E-70 |
| complement(30285..31475) |  | PHAGE_Salmon_SEN5_NC_028701: tail sheath monomer; PP_00042; phage(gi966201583) | 0 |
| 31625..32749 |  | PHAGE_Salmon_SEN5_NC_028701: late control D protein; PP_00043; phage(gi966201585) | 1.03E-171 |
| 32800..33051 |  | PHAGE_Entero_WPhi_NC_005056: Ogr; PP_00044; phage(gi33438926) | 2.23E-15 |
| complement(33091..33615) |  | hypothetical; PP_00045 | 0 |
| 33813..33840 |  | attR | 0 |
| complement(33937..36462) |  | PROPHAGE_Shigel_301: serine protease; PP_00046; phage(gi24114232) | 8.34E-17 |
|  |  |  |  |
| **Region 2 (20.5kb, 39.64%)** | **Incomplete** | **PHAGE_Salmon_118970_sal4_NC_030919** |  |
| 1537841..1537888 |  | attL | 0 |
| complement(1537908..1538051) |  | PHAGE_Salmon_SEN22_NC_028696: integrase; PP_01483; phage(gi966201337) | 1.33E-12 |
| complement(1538054..1538587) |  | PHAGE_Salmon_118970_sal4_NC_030919: hypothetical protein; PP_01484; phage(gi100004) | 1.18E-89 |
| 1539029..1539184 |  | hypothetical; PP_01485 | 0 |
| 1539615..1540145 |  | PHAGE_Entero_phi80_NC_021190: DNA junction-specific endonuclease Rap; PP_01486; phage(gi824479632) | 2.98E-70 |
| 1540447..1540659 |  | PHAGE_Lactoc_bIL312_NC_002671: Csp; PP_01487; phage(gi13095918) | 3.34E-20 |
| 1540990..1541694 |  | hypothetical; PP_01488 | 0 |
| 1541766..1542419 |  | hypothetical; PP_01489 | 0 |
| 1543503..1543961 |  | PHAGE_Pelagi_HTVC008M_NC_020484: heat shock protein; PP_01490; phage(gi460042547) | 1.29E-15 |
| complement(1545286..1545561) |  | PHAGE_Edward_GF_2_NC_026611: hypothetical protein; PP_01491; phage(gi764162266) | 7.70E-06 |
| 1545946..1546059 |  | PHAGE_Salmon_118970_sal4_NC_030919: hypothetical protein; PP_01492; phage(gi100038) | 4.88E-09 |
| 1546052..1546528 |  | PHAGE_Salmon_SE1_NC_011802: Gp19; PP_01493; phage(gi219681236) | 1.72E-67 |
| 1546528..1546668 |  | hypothetical; PP_01494 | 0 |
| 1546665..1547042 |  | hypothetical; PP_01495 | 0 |
| 1547011..1547184 |  | PHAGE_Entero_SfV_NC_003444: putative Rz1 lytic protein; PP_01496; phage(gi19549039) | 1.75E-06 |
| 1547283..1547444 |  | hypothetical; PP_01497 | 0 |
| 1547720..1547971 |  | hypothetical; PP_01498 | 0 |
| 1548066..1548983 |  | PHAGE_Entero_YYZ_2008_NC_011356: putative major head protein/prohead proteinase; PP_01499; phage(gi209427776) | 6.73E-117 |
| 1549422..1549535 |  | PHAGE_Phage_Gifsy_2_NC_010393: hypothetical protein; PP_01500; phage(gi169257293) | 9.19E-06 |
| complement(1549624..1549959) |  | hypothetical; PP_01501 | 0 |
| complement(1550275..1551711) |  | hypothetical; PP_01502 | 0 |
| complement(1551726..1553849) |  | PHAGE_Bacill_SPbeta_NC_001884: ABC transporter; PP_01503; phage(gi9630145) | 8.72E-50 |
| 1558428..1558475 |  | attR | 0 |
|  |  |  |  |
| **Region 3 (12.9kb, 44.49%)** | **Incomplete** | **PHAGE_Entero_P4_NC_001609** |  |
| 2258564..2258586 |  | attL | 0 |
| 2258897..2259931 |  | PROPHAGE_Escher_Sakai: putative integrase; PP_02234; phage(gi15833788) | 1.77E-156 |
| complement(2259915..2260847) |  | PHAGE_Burkho_KS5_NC_015265: gp44; PP_02235; phage(gi327198042) | 1.25E-06 |
| complement(2260849..2261832) |  | hypothetical; PP_02236 | 0 |
| complement(2263047..2265764) |  | PHAGE_Entero_P4_NC_001609: DNA primase; PP_02237; phage(gi9627512) | 5.34E-36 |
| complement(2265751..2265990) |  | hypothetical; PP_02238 | 0 |
| complement(2265987..2266184) |  | PHAGE_Entero_P4_NC_001609: putative CI repressor; PP_02239; phage(gi9627516) | 2.20E-09 |
| complement(2266580..2267101) |  | PHAGE_Aeromo_phiO18P_NC_009542: capsid-scaffolding protein; PP_02240; phage(gi148727152) | 4.67E-41 |
| complement(2267098..2268117) |  | PHAGE_Aeromo_phiO18P_NC_009542: capsid protein; PP_02241; phage(gi148727153) | 1.30E-70 |
| complement(2268147..2268989) |  | hypothetical; PP_02242 | 0 |
| 2269716..2269738 |  | attR | 0 |
| complement(2269763..2269891) |  | hypothetical; PP_02243 | 0 |
| complement(2270095..2270313) |  | hypothetical; PP_02244 | 0 |
| complement(2270367..2270558) |  | PHAGE_Entero_SfV_NC_003444: putative Rz1 lytic protein; PP_02245; phage(gi19549039) | 2.38E-08 |
| complement(2270530..2270907) |  | hypothetical; PP_02246 | 0 |
| complement(2270904..2271044) |  | hypothetical; PP_02247 | 0 |
| complement(2271044..2271517) |  | PHAGE_Salmon_SE1_NC_011802: Gp19; PP_02248; phage(gi219681236) | 7.14E-60 |
|  |  |  |  |
| **Region 4 (40.5kb, 48.49%)** | **Intact** | **PHAGE_Entero_mEp235_NC_019708** |  |
| 2287625..2287649 |  | attL | 0 |
| 2287661..2288059 |  | PHAGE_Entero_mEp235_NC_019708: integrase; PP_02277; phage(gi428781836) | 2.57E-22 |
| complement(2289335..2289499) |  | hypothetical; PP_02278 | 0 |
| complement(2289518..2289643) |  | hypothetical; PP_02279 | 0 |
| complement(2289754..2291016) |  | PHAGE_Salmon_vB_SosS_Oslo_NC_018279: error-prone lesion bypass DNA polymerase V; PP_02280; phage(gi399528790) | 1.72E-175 |
| complement(2291016..2291255) |  | PHAGE_Salmon_vB_SosS_Oslo_NC_018279: errror-prone repair protein UmuD; PP_02281; phage(gi399528789) | 9.58E-27 |
| complement(2292335..2293921) |  | PHAGE_Entero_mEp237_NC_019704: tail fiber; PP_02282; phage(gi435439289) | 9.34E-39 |
| complement(2293975..2294658) |  | hypothetical; PP_02283 | 0 |
| complement(2294900..2295181) |  | hypothetical; PP_02284 | 0 |
| complement(2295187..2298366) |  | PHAGE_Entero_HK225_NC_019717: central tail fiber; PP_02285; phage(gi428782397) | 0 |
| complement(2298399..2299007) |  | PHAGE_Entero_mEp234_NC_019715: tail assembly protein I; PP_02286; phage(gi428782273) | 4.81E-92 |
| complement(2299992..2300699) |  | PHAGE_Entero_mEp235_NC_019708: minor tail protein K; PP_02287; phage(gi428781828) | 7.61E-125 |
| complement(2300702..2301460) |  | PHAGE_Entero_mEp235_NC_019708: minor tail protein L; PP_02288; phage(gi428781827) | 3.10E-137 |
| complement(2301457..2301792) |  | PHAGE_Entero_HK446_NC_019714: minor tail protein M; PP_02289; phage(gi428782208) | 4.26E-51 |
| complement(2301789..2305049) |  | PHAGE_Entero_mEp390_NC_019721: tail length tape measure protein; PP_02290; phage(gi428782677) | 0 |
| complement(2305075..2305359) |  | PHAGE_Entero_HK106_NC_019768: tail protein; PP_02291; phage(gi428783291) | 6.20E-29 |
| complement(2305371..2305754) |  | PHAGE_Escher_HK75_NC_016160: tail assembly chaperone; PP_02292; phage(gi356870688) | 2.76E-43 |
| complement(2305758..2306225) |  | PHAGE_Entero_mEp234_NC_019715: major tail subunit; PP_02293; phage(gi428782264) | 5.19E-59 |
| complement(2306285..2306620) |  | PHAGE_Entero_mEp234_NC_019715: hypothetical protein; PP_02294; phage(gi428782263) | 1.45E-31 |
| complement(2306617..2307066) |  | PHAGE_Escher_HK75_NC_016160: hypothetical protein; PP_02295; phage(gi356870685) | 4.55E-56 |
| complement(2307059..2307280) |  | PHAGE_Entero_mEp235_NC_019708: hypothetical protein; PP_02296; phage(gi428781819) | 6.26E-15 |
| complement(2307396..2307689) |  | PHAGE_Entero_mEp235_NC_019708: head-tail connector II; PP_02297; phage(gi428781817) | 5.21E-06 |
| complement(2307731..2308942) |  | PHAGE_Entero_SfI_NC_027339: phage major capsid protein; PP_02298; phage(gi849250290) | 0 |
| complement(2308952..2309599) |  | PHAGE_Salmon_ST64B_NC_004313: Pro-head protease; PP_02299; phage(gi23505450) | 5.09E-123 |
| complement(2309592..2310812) |  | PHAGE_Entero_phiP27_NC_003356: putative portal protein; PP_02300; phage(gi18249902) | 0 |
| complement(2310812..2310991) |  | PHAGE_Klebsi_phiKO2_NC_005857: Gp3; PP_02301; phage(gi46402089) | 2.68E-15 |
| complement(2311001..2312731) |  | PHAGE_Entero_mEp235_NC_019708: terminase large subunit; PP_02302; phage(gi428781812) | 0 |
| complement(2312735..2313205) |  | PHAGE_Entero_mEp235_NC_019708: terminase small subunit; PP_02303; phage(gi428781811) | 1.27E-80 |
| 2313439..2313699 |  | hypothetical; PP_02304 | 0 |
| 2313674..2313799 |  | hypothetical; PP_02305 | 0 |
| complement(2313814..2314329) |  | hypothetical; PP_02306 | 0 |
| complement(2314949..2315326) |  | PHAGE_Endosy_APSE_1_NC_000935: hypothetical protein; PP_02307; phage(gi9633561) | 2.54E-07 |
| complement(2315323..2315481) |  | hypothetical; PP_02308 | 0 |
| complement(2315463..2315939) |  | PHAGE_Salmon_SE1_NC_011802: Gp19; PP_02309; phage(gi219681236) | 7.08E-64 |
| complement(2315932..2316123) |  | PHAGE_Bacter_APSE_2_NC_011551: phage 21-like group II holin; PP_02310; phage(gi212499716) | 5.94E-19 |
| 2328179..2328203 |  | attR | 0 |
|  |  |  |  |
| **Region 5 (45.2kb, 49.68%)** | **Intact** | **PHAGE_Entero_mEp460_NC_019716** |  |
| 2413042..2413095 |  | attL | 0 |
| complement(2413765..2415345) |  | PHAGE_Salmon_vB_SosS_Oslo_NC_018279: minor tail protein; PP_02415; phage(gi399528788) | 5.96E-39 |
| complement(2415458..2415724) |  | hypothetical; PP_02416 | 0 |
| complement(2415727..2416413) |  | hypothetical; PP_02417 | 0 |
| complement(2416732..2419896) |  | PHAGE_Entero_BP_4795_NC_004813: putative tail component; PP_02418; phage(gi157166057) | 0 |
| complement(2419909..2420487) |  | PHAGE_Entero_mEp460_NC_019716: tail assembly protein; PP_02419; phage(gi428782333) | 3.06E-61 |
| complement(2420457..2421185) |  | PHAGE_Entero_mEp460_NC_019716: tail fiber component; PP_02420; phage(gi428782332) | 8.12E-105 |
| complement(2421203..2421901) |  | PHAGE_Entero_mEp460_NC_019716: minor tail protein; PP_02421; phage(gi428782331) | 2.34E-83 |
| complement(2421914..2422198) |  | PHAGE_Acinet_AP22_NC_017984: putative DNA-binding protein; PP_02422; phage(gi388570841) | 5.79E-06 |
| complement(2422681..2422806) |  | hypothetical; PP_02423 | 0 |
| complement(2423321..2426248) |  | PHAGE_Entero_mEp460_NC_019716: tail length tape measure protein; PP_02424; phage(gi428782329) | 5.87E-134 |
| complement(2426229..2426495) |  | PHAGE_Entero_mEp460_NC_019716: tail assembly protein; PP_02425; phage(gi428782328) | 9.54E-19 |
| complement(2426561..2426962) |  | PHAGE_Entero_mEp460_NC_019716: minor tail protein; PP_02426; phage(gi428782327) | 1.52E-20 |
| complement(2426978..2427493) |  | PHAGE_Entero_phi80_NC_021190: major tail protein; PP_02427; phage(gi824479592) | 1.52E-81 |
| complement(2427503..2427901) |  | PHAGE_Entero_c_1_NC_019706: tail protein; PP_02428; phage(gi428781744) | 2.56E-56 |
| complement(2427901..2428464) |  | PHAGE_Entero_HK225_NC_019717: minor tail protein Z; PP_02429; phage(gi428782386) | 8.83E-72 |
| complement(2428466..2428744) |  | PHAGE_Entero_mEp460_NC_019716: hypothetical protein; PP_02430; phage(gi428782323) | 1.49E-20 |
| complement(2428749..2429090) |  | PHAGE_Entero_mEp460_NC_019716: hypothetical protein; PP_02431; phage(gi428782322) | 7.63E-21 |
| complement(2429176..2431212) |  | PHAGE_Entero_mEp460_NC_019716: putative protease/scaffold protein; PP_02432; phage(gi428782321) | 0 |
| complement(2431184..2432659) |  | PHAGE_Entero_mEp460_NC_019716: portal protein; PP_02433; phage(gi428782320) | 0 |
| complement(2432656..2432874) |  | PHAGE_Entero_mEp460_NC_019716: hypothetical protein; PP_02434; phage(gi428782319) | 4.06E-24 |
| complement(2432871..2434985) |  | PHAGE_Entero_mEp460_NC_019716: terminase large subunit; PP_02435; phage(gi428782318) | 0 |
| complement(2434982..2435485) |  | PHAGE_Entero_mEp460_NC_019716: terminase small subunit; PP_02436; phage(gi428782317) | 6.82E-22 |
| 2435585..2435737 |  | hypothetical; PP_02437 | 0 |
| complement(2435809..2435994) |  | PHAGE_Entero_phiP27_NC_003356: hypothetical protein; PP_02438; phage(gi18249897) | 1.65E-16 |
| complement(2436631..2437008) |  | hypothetical; PP_02439 | 0 |
| complement(2437005..2437145) |  | hypothetical; PP_02440 | 0 |
| complement(2437145..2437621) |  | PHAGE_Salmon_SE1_NC_011802: Gp19; PP_02441; phage(gi219681236) | 6.21E-64 |
| complement(2437614..2437805) |  | PHAGE_Bacter_APSE_2_NC_011551: phage 21-like group II holin; PP_02442; phage(gi212499716) | 7.51E-19 |
| complement(2438043..2439062) |  | hypothetical; PP_02443 | 0 |
| complement(2439488..2439601) |  | hypothetical; PP_02444 | 0 |
| complement(2439607..2440662) |  | PHAGE_Entero_mEp460_NC_019716: DNA methylase; PP_02445; phage(gi428782369) | 2.81E-169 |
| complement(2440801..2440995) |  | PHAGE_Entero_mEp460_NC_019716: hypothetical protein; PP_02446; phage(gi428782368) | 2.02E-09 |
| complement(2441161..2441838) |  | PHAGE_Phage_Gifsy_1_NC_010392: bacteriophage antiterminator protein Q; PP_02447; phage(gi169257244) | 2.60E-34 |
| complement(2441869..2442885) |  | PHAGE_Entero_mEp460_NC_019716: hypothetical protein; PP_02448; phage(gi428782365) | 6.65E-70 |
| complement(2442885..2443673) |  | PHAGE_Shigel_SfII_NC_021857: KliA-N domain protein; PP_02449; phage(gi526244680) | 1.66E-116 |
| complement(2443691..2444098) |  | PHAGE_Entero_mEp460_NC_019716: holliday junction resolvase; PP_02450; phage(gi428782363) | 1.80E-42 |
| complement(2444283..2444678) |  | hypothetical; PP_02451 | 0 |
| complement(2444678..2445814) |  | PHAGE_Salmon_9NA_NC_025443: putative DNA methylase; PP_02452; phage(gi712914017) | 7.90E-105 |
| complement(2445814..2446062) |  | hypothetical; PP_02453 | 0 |
| complement(2446059..2446943) |  | PHAGE_Entero_mEp460_NC_019716: replication protein; PP_02454; phage(gi428782359) | 3.23E-34 |
| complement(2446940..2447131) |  | PHAGE_Salmon_118970_sal3_NC_031940: hypothetical protein; PP_02455; phage(gi100119) | 7.76E-06 |
| complement(2447124..2447324) |  | hypothetical; PP_02456 | 0 |
| complement(2447388..2447867) |  | PHAGE_Entero_mEp390_NC_019721: hypothetical protein; PP_02457; phage(gi428782704) | 9.97E-40 |
| complement(2448959..2449846) |  | PHAGE_Vibrio_K139_NC_003313: hypothetical protein; PP_02458; phage(gi17975108) | 1.52E-40 |
| 2449998..2450210 |  | hypothetical; PP_02459 | 0 |
| 2450431..2450793 |  | PHAGE_Salmon_ST64B_NC_004313: hypothetical protein; PP_02460; phage(gi23505481) | 1.69E-46 |
| 2450859..2451686 |  | PHAGE_Salmon_118970_sal3_NC_031940: rI antiholin; PP_02461; phage(gi100112) | 1.06E-103 |
| 2451796..2452320 |  | PHAGE_Entero_mEp460_NC_019716: hypothetical protein; PP_02462; phage(gi428782349) | 6.34E-66 |
| 2452514..2453026 |  | hypothetical; PP_02463 | 0 |
| 2453089..2453502 |  | PHAGE_Pectob_ZF40_NC_019522: putative methylase; PP_02464; phage(gi422936660) | 5.04E-59 |
| 2453495..2454064 |  | PHAGE_Entero_mEp460_NC_019716: putative exonuclease; PP_02465; phage(gi428782342) | 2.26E-50 |
| 2454312..2455484 |  | PHAGE_Entero_HK022_NC_002166: integrase; PP_02466; phage(gi9634144) | 1.47E-17 |
| 2455618..2455671 |  | attR | 0 |
| complement(2455729..2456316) |  | hypothetical; PP_02467 | 0 |
| 2456435..2457661 |  | PHAGE_Lactob_phiAQ113_NC_019782: phosphoadenosine phosphosulfate reductase; PP_02468; phage(gi446730264) | 4.53E-72 |
| 2457680..2458309 |  | PHAGE_Mycoba_Gaia_NC_026590: ectoine synthase/ParB multidomain protein; PP_02469; phage(gi764160185) | 1.33E-46 |
|  |  |  |  |

^†^The completeness of prophage regions was determined by the scores calculated using PHASTER (<http://phaster.ca/>). [Intact (score > 90); Questionable (score 70-90); Incomplete (score < 70)].
