# Supplementary material for: Emergence of Third-Generation Cephalosporin-Resistant Morganella morganii in a Captive Breeding Dolphin in South Korea
Source: Animals (Basel). 2020 Nov 6;10(11):2052. doi: 10.3390/ani10112052 (PMC7694518; doi:10.3390/ani10112052)
Supplement: Supplementary file 1 [file animals-10-02052-s001.zip › Supplements (Mm_animals)/Table S2. Mm(Fv).docx]

**Table S2**. Potential virulence genes detected in *Morganella morganii* KC-Tt-01.

| **No** | **Predicted genes** | **Description** | **Identity**  **(%)** | **Alignment length** | **Mis-matches** | **Gap** | **QSS^†^** | **QSE^†^** | **DSS^†^** | **DSE^†^** | **e-value** | **Bit score** |
| --- | --- | --- | --- | --- | --- | --- | --- | --- | --- | --- | --- | --- |
| 1 | elongation factor Tu | *Morganella morganii* subsp. *morganii* KT | 98.7 | 1185 | 15 | 0 | 160596 | 161780 | 750462 | 751646 | 0 | 2069 |
| 2 | methyl-accepting chemotaxis protein | *Morganella morganii* subsp. *morganii* KT | 98.4 | 1578 | 25 | 0 | 185301 | 186878 | 775167 | 776744 | 0 | 2776 |
| 3 | glucose-6-phosphate isomerase | *Morganella morganii* subsp. *morganii* KT | 98.1 | 1647 | 34 | 0 | 216885 | 218531 | 805269 | 806915 | 0 | 2854 |
| 4 | urease subunit gamma | *Morganella morganii* subsp. *morganii* KT | 100 | 303 | 0 | 0 | 321100 | 321402 | 909328 | 909630 | 0 | 560 |
| 5 | urease subunit beta | *Morganella morganii* subsp. *morganii* KT | 99.3 | 423 | 3 | 0 | 321432 | 321854 | 909660 | 910082 | 0 | 765 |
| 6 | urease subunit alpha | *Morganella morganii* subsp. *morganii* KT | 98.1 | 1719 | 32 | 0 | 321922 | 323640 | 910150 | 911868 | 0 | 2998 |
| 7 | urease accessory protein UreE | *Morganella morganii* subsp. *morganii* KT | 97.7 | 684 | 16 | 12 | 323697 | 324380 | 911925 | 912596 | 0 | 1164 |
| 8 | urease accessory protein UreF | *Morganella morganii* subsp. *morganii* KT | 99.9 | 687 | 1 | 0 | 324405 | 325091 | 912621 | 913307 | 0 | 1264 |
| 9 | urease accessory protein UreG | *Morganella morganii* subsp. *morganii* KT | 99.4 | 636 | 4 | 0 | 325125 | 325760 | 913341 | 913976 | 0 | 1153 |
| 10 | urea transporter | *Morganella morganii* subsp. *morganii* KT | 98.8 | 993 | 12 | 0 | 326816 | 327808 | 915032 | 916024 | 0 | 1768 |
| 11 | RNA polymerase sigma factor RpoS | *Morganella morganii* subsp. *morganii* KT | 99.1 | 993 | 9 | 0 | 513533 | 514525 | 1101680 | 1102672 | 0 | 1784 |
| 12 | ABC transporter ATP-binding protein^‡^ | *Morganella morganii* GN28 | 99.5 | 1728 | 9 | 0 | 830739 | 832466 | 1005115 | 1003388 | 0 | 3142 |
| 13 | non-ribosomal peptide synthetase^‡^ | *Morganella morganii* GN28 | 99.6 | 6105 | 27 | 0 | 845331 | 851435 | 1025424 | 1019320 | 0 | 11125 |
| 14 | gmhA | *Morganella morganii* subsp. *morganii* KT | 99.3 | 579 | 4 | 0 | 882200 | 882778 | 1443046 | 1443624 | 0 | 1048 |
| 15 | ferric iron uptake transcriptional regulator | *Morganella morganii* subsp. *morganii* KT | 100 | 450 | 0 | 0 | 1005517 | 1005966 | 1566562 | 1566113 | 0 | 832 |
| 16 | porin OmpC | *Morganella morganii* subsp. *morganii* KT | 99.7 | 1101 | 3 | 0 | 1204705 | 1205805 | 1767131 | 1766031 | 0 | 2017 |
| 17 | porin OmpA | *Morganella morganii* subsp. *morganii* KT | 100 | 1074 | 0 | 0 | 1228027 | 1229100 | 1790412 | 1789339 | 0 | 1984 |
| 18 | flagellar transcriptional regulator FlhD | *Morganella morganii* subsp. *morganii* KT | 100 | 354 | 0 | 0 | 1434778 | 1435131 | 1987618 | 1987971 | 0 | 654 |
| 19 | flagellar transcriptional regulator FlhC | *Morganella morganii* subsp. *morganii* KT | 99.7 | 582 | 2 | 0 | 1435137 | 1435718 | 1987977 | 1988558 | 0 | 1064 |
| 20 | flagellar motor stator protein MotA | *Morganella morganii* subsp. *morganii* KT | 99.2 | 888 | 7 | 0 | 1435818 | 1436705 | 1988658 | 1989545 | 0 | 1602 |
| 21 | motility protein MotB | *Morganella morganii* subsp. *morganii* KT | 97.8 | 1002 | 22 | 0 | 1436713 | 1437714 | 1989553 | 1990554 | 0 | 1729 |
| 22 | chemotaxis protein CheA | *Morganella morganii* subsp. *morganii* KT | 99.6 | 2073 | 8 | 0 | 1437791 | 1439863 | 1990631 | 1992703 | 0 | 3784 |
| 23 | methyl-accepting chemotaxis protein | *Morganella morganii* subsp. *morganii* KT | 99.9 | 1686 | 1 | 0 | 1440473 | 1442158 | 1993313 | 1994998 | 0 | 3109 |
| 24 | chemotaxis response regulator protein-glutamate methylesterase | *Morganella morganii* subsp. *morganii* KT | 98.8 | 1068 | 13 | 0 | 1444800 | 1445867 | 1997640 | 1998707 | 0 | 1901 |
| 25 | flagellar type III secretion system protein FlhB | *Morganella morganii* subsp. *morganii* KT | 99 | 1152 | 12 | 0 | 1447673 | 1448824 | 2000513 | 2001664 | 0 | 2061 |
| 26 | flagellar biosynthesis protein FlhA | *Morganella morganii* subsp. *morganii* KT | 99 | 2097 | 21 | 0 | 1448817 | 1450913 | 2001657 | 2003753 | 0 | 3757 |
| 27 | insecticidal toxin protein | *Morganella morganii* subsp. *morganii* KT | 96.3 | 2235 | 82 | 2 | 1455324 | 1462835 | 2008516 | 2006283 | 0 | 3672 |
| 28 | flagellar hook protein FlgE | *Morganella morganii* subsp. *morganii* KT | 99.9 | 1212 | 1 | 0 | 1466681 | 1467892 | 2013696 | 2014907 | 0 | 2233 |
| 29 | flagellar basal body rod protein FlgF | *Morganella morganii* subsp. *morganii* KT | 99.2 | 756 | 6 | 0 | 1467914 | 1468669 | 2014929 | 2015684 | 0 | 1363 |
| 30 | flagellar basal body L-ring protein FlgH | *Morganella morganii* subsp. *morganii* KT | 99.2 | 759 | 6 | 0 | 1469548 | 1470297 | 2016563 | 2017312 | 0 | 1352 |
| 31 | flagellar basal body P-ring protein FlgI | *Morganella morganii* subsp. *morganii* KT | 99.3 | 1107 | 8 | 0 | 1470315 | 1471421 | 2017330 | 2018436 | 0 | 2001 |
| 32 | flagellar biosynthetic protein FliP | *Morganella morganii* subsp. *morganii* KT | 100 | 741 | 0 | 0 | 1478286 | 1479026 | 2026041 | 2025301 | 0 | 1369 |
| 33 | flagellar motor switch protein FliN | *Morganella morganii* subsp. *morganii* KT | 100 | 411 | 0 | 0 | 1479504 | 1479914 | 2026929 | 2026519 | 0 | 760 |
| 34 | flagellum-specific ATP synthase FliI | *Morganella morganii* subsp. *morganii* KT | 98.7 | 1365 | 18 | 0 | 1483398 | 1484762 | 2031777 | 2030413 | 0 | 2422 |
| 35 | flagellar motor switch protein FliG | *Morganella morganii* subsp. *morganii* KT | 99.5 | 993 | 5 | 0 | 1485483 | 1486475 | 2033490 | 2032498 | 0 | 1807 |
| 36 | flagellin FliC | *Morganella morganii* subsp. *morganii* KT | 100 | 1152 | 0 | 0 | 1495529 | 1496680 | 2042543 | 2043694 | 0 | 2128 |
| 37 | flagellin FliA | *Morganella morganii* subsp. *morganii* KT | 100 | 1071 | 0 | 0 | 1496911 | 1497981 | 2043925 | 2044995 | 0 | 1978 |
| 38 | HlyD family type I secretion periplasmic adaptor subunit^‡^ | *Morganella morganii* GN28 | 99.9 | 1437 | 2 | 0 | 1550275 | 1551711 | 2773176 | 2774612 | 0 | 2643 |
| 39 | RTX toxin hemolysin A^‡^ | *Morganella morganii* FDAARGOS_172 | 99.1 | 3093 | 27 | 0 | 1553921 | 1557013 | 1063143 | 1060069 | 0 | 5546 |
| 40 | toxin-activating lysine-acyltransferase^‡^ | *Morganella morganii* FDAARGOS_63 | 100 | 513 | 0 | 0 | 1557026 | 1557538 | 2828247 | 2827735 | 0 | 948 |
| 41 | magnesium-translocating P-type ATPase | *Morganella morganii* subsp. *morganii* KT | 98.4 | 2697 | 43 | 0 | 1776003 | 1778699 | 2374507 | 2377203 | 0 | 4743 |
| 42 | catalase | *Morganella morganii* subsp. *morganii* KT | 98.6 | 1443 | 20 | 0 | 2180767 | 2182209 | 2781129 | 2782571 | 0 | 2555 |
| 43 | two-component system response regulator PhoP | *Morganella morganii* subsp. *morganii* KT | 97.6 | 675 | 16 | 0 | 2338571 | 2339245 | 2929739 | 2930413 | 0 | 1158 |
| 44 | 3-oxoacyl-ACP reductase FabG | *Morganella morganii* subsp. *morganii* KT | 99.9 | 735 | 1 | 0 | 2369734 | 2370468 | 2961633 | 2960899 | 0 | 1352 |
| 45 | TonB-dependent receptor | *Morganella morganii* subsp. *morganii* KT | 98.1 | 2040 | 39 | 0 | 2541076 | 2543115 | 3162290 | 3160251 | 0 | 3552 |
| 46 | type IV secretion protein_Rhs | *Morganella morganii* subsp. *morganii* KT | 96.3 | 2235 | 83 | 19 | 2806161 | 2808836 | 3429922 | 3427698 | 0 | 3650 |
| 47 | RNA polymerase sigma factor RpoD | *Morganella morganii* subsp. *morganii* KT | 99.6 | 1851 | 8 | 0 | 2916776 | 2918626 | 3534234 | 3536084 | 0 | 3374 |
| 48 | methyl-accepting chemotaxis protein | *Morganella morganii* subsp. *morganii* KT | 98 | 1562 | 31 | 0 | 3032733 | 3034298 | 3644318 | 3645879 | 0 | 2713 |
| 49 | PapC/FimD family outer membrane usher protein | *Morganella morganii* subsp. *morganii* KT | 99.3 | 2658 | 19 | 0 | 3173595 | 3176252 | 3757606 | 3754949 | 0 | 4804 |
| 50 | fimbria A protein | *Morganella morganii* subsp. *morganii* KT | 99.9 | 528 | 1 | 0 | 3176901 | 3177428 | 3758782 | 3758255 | 0 | 970 |
| 51 | porin | *Morganella morganii* subsp. *morganii* KT | 97.4 | 1151 | 30 | 7 | 3180203 | 3181348 | 3762704 | 3761556 | 0 | 1953 |
| 52 | histidine-histamine antiporter (hdcT1) | *Morganella morganii* subsp. *morganii* KT | 99.9 | 1362 | 5 | 0 | 3210810 | 3212171 | 3792166 | 3793527 | 0 | 2488 |
| 53 | histidine decarboxylase (hdc) | *Morganella morganii* subsp. *morganii* KT | 99.9 | 1137 | 4 | 0 | 3212222 | 3213358 | 3793578 | 3794714 | 0 | 2078 |
| 54 | histidine/histamine antiporter (hdcT2) | *Morganella morganii* subsp. *morganii* KT | 99.9 | 1413 | 5 | 0 | 3213592 | 3215004 | 3794948 | 3796360 | 0 | 2582 |
| 55 | histidyl-tRNA synthetase (hisRS) | *Morganella morganii* subsp. *morganii* KT | 99.9 | 1272 | 2 | 0 | 3215015 | 3216286 | 3796371 | 3797642 | 0 | 2338 |
| 56 | EscV/YscV/HrcV family type III secretion system export apparatus protein | *Morganella morganii* subsp. *morganii* KT | 99.5 | 2061 | 10 | 0 | 3251191 | 3253251 | 35045 | 32985 | 0 | 3751 |
| 57 | nitrate reductase subunit beta | *Morganella morganii* subsp. *morganii* KT | 99.2 | 1557 | 12 | 0 | 3294658 | 3296214 | 77314 | 78870 | 0 | 2809 |
| 58 | TonB-dependent siderophore receptor | *Morganella morganii* subsp. *morganii* KT | 98.7 | 2214 | 28 | 0 | 3324650 | 3326863 | 109613 | 107400 | 0 | 3934 |
| 59 | lipoprotein NlpI | *Morganella morganii* subsp. *morganii* KT | 99.9 | 888 | 1 | 0 | 3396772 | 3397659 | 186139 | 185252 | 0 | 1635 |
| 60 | phosphoglucosamine mutase | *Morganella morganii* subsp. *morganii* KT | 97.6 | 1335 | 32 | 0 | 3407810 | 3409144 | 197624 | 196290 | 0 | 2289 |
| 61 | glucose-1-phosphate thymidylyltransferase | *Morganella morganii* subsp. *morganii* KT | 100 | 882 | 0 | 0 | 3491005 | 3491886 | 280368 | 279487 | 0 | 1629 |
| 62 | elongation factor Tu | *Morganella morganii* subsp. *morganii* KT | 98.6 | 1185 | 16 | 0 | 3548192 | 3549376 | 337848 | 336664 | 0 | 2100 |
| 63 | ADP-glyceromanno-heptose 6-epimerase | *Morganella morganii* subsp. *morganii* KT | 97.7 | 939 | 22 | 2 | 3805985 | 3806923 | 603668 | 602730 | 0 | 1613 |
| 64 | polysaccharide biosynthesis protein | *Morganella morganii* subsp. *morganii* KT | 100 | 1863 | 0 | 0 | 3815199 | 3817061 | 3817061 | 3815199 | 0 | 3441 |

**^†^**QSS, Query sequence start; QSE, Query sequence end; DSS, Database sequence start; DSE, Database sequence end.

^‡^Virulence genes not detected on *M. morganii* subsp. *morganii* KT.
