# Supplementary material for: Emergence of Third-Generation Cephalosporin-Resistant Morganella morganii in a Captive Breeding Dolphin in South Korea
Source: Animals (Basel). 2020 Nov 6;10(11):2052. doi: 10.3390/ani10112052 (PMC7694518; doi:10.3390/ani10112052)
Supplement: Supplementary file 1 [file animals-10-02052-s001.zip › Supplements (Mm_animals)/Table S3. Mm(Fv).docx]

**Table S3**. Potential antimicrobial resistance genes detected in *Morganella morganii* KC-Tt-01.

| **No** | **Resistance gene** | **Description** | **Identity (%)** | **Alignment length** | **Mis-matches** | **Gap** | **QSS^†^** | **QSE^†^** | **DSS^†^** | **DSE^†^** | **e-value** | **Bit score** |
| --- | --- | --- | --- | --- | --- | --- | --- | --- | --- | --- | --- | --- |
| 1 | Multidrug efflux MFS transporter subunit emrb | *Morganella morganii* GN28 | 99.9 | 1533 | 1 | 0 | 950147 | 951679 | 3449950 | 3451482 | 0 | 2760 |
| 2 | Multidrug export protein emra | *Morganella morganii* FDAARGOS_63 | 99.9 | 1179 | 1 | 0 | 951692 | 952870 | 2151329 | 2150151 | 0 | 2122 |
| 3 | Penicillin-binding protein 2 | *Morganella morganii* AR_0057 | 99.5 | 1879 | 10 | 0 | 972649 | 974529 | 2018360 | 2016482 | 0 | 3415 |
| 4 | Bifunctional UDP-4-amino-4-deoxy-L-arabinose formyltransferase/UDP-glucuronic acid oxidase arna | *Morganella morganii* AR_0057 | 99.8 | 1986 | 3 | 0 | 2068012 | 2069997 | 3303472 | 3301487 | 0 | 3568 |
| 5 | Type A chloramphenicol O-acetyltransferase cata2 | *Morganella morganii* FDAARGOS_172 | 99.8 | 642 | 1 | 0 | 2523168 | 2523809 | 2050209 | 2049568 | 0 | 1181 |
| 6 | 1,6-anhydro-N-acetylmuramyl-L-alanine amidase ampd | *Morganella morganii* FDAARGOS_365 | 100 | 560 | 0 | 0 | 2622368 | 2622928 | 711586 | 712145 | 0 | 1011 |
| 7 | Multidrug efflux MFS transporter subunit emrb | *Morganella morganii* GN28 | 99.5 | 1521 | 8 | 0 | 2702267 | 2703787 | 1564818 | 1566338 | 0 | 2708 |
| 8 | Emra/emrk family multidrug efflux transporter periplasmic adaptor subunit | *Morganella morganii* FDAARGOS_365 | 100 | 1191 | 0 | 0 | 2703803 | 2704993 | 629527 | 630717 | 0 | 2149 |
| 9 | Class C beta-lactamase DHA-4 | *Morganella morganii* AR_0057 | 98.9 | 1140 | 11 | 0 | 2938485 | 2939624 | 131829 | 130690 | 0 | 2106 |
| 10 | ampR | *Morganella morganii* AR_0057 | 98.7 | 876 | 11 | 0 | 2939735 | 2940610 | 131940 | 132815 | 0 | 1557 |
| 11 | D-alanyl-D-alanine-carboxypeptidase/endopeptidase amph | *Morganella morganii* AR_0057 | 99.3 | 1224 | 9 | 0 | 3362328 | 3363551 | 1051664 | 1050441 | 0 | 2211 |

**^†^**QSS, Query sequence start; QSE, Query sequence end; DSS, Database sequence start; DSE, Database sequence end.
